# Diagnostic Accuracy of Intestinal Ultrasound for Detecting Postoperative Recurrence in Crohn’s Disease: A Systematic Review

**DOI:** 10.7759/cureus.108712

**Published:** 2026-05-12

**Authors:** Jalal Abu Halimah, Abdulla H Alkhalifa, Renad H Alqurashi, Faisal B Alahmadi, Sarah A Almousa, Fatimah M Qarn, Fouz M Alshamrani, Fay N Alanazi, Mohammed A Balhareth, Abdulmaged M Alghthy, Ruba M Almuallim, Bushra S Albakri

**Affiliations:** 1 Department of Surgery, Faculty of Medicine, Jazan University, Jazan, SAU; 2 College of Medicine, King Faisal University, Al Ahsa, SAU; 3 College of Medicine, Taif University, Taif, SAU; 4 Faculty of Medicine, Taibah University, Madinah, SAU; 5 Faculty of Medicine, Jazan University, Jazan, SAU; 6 Faculty of Medicine, University of Tabuk, Tabuk, SAU; 7 College of Medicine, Tabuk University, Tabuk, SAU; 8 Faculty of Medicine, King Faisal University, Al Ahsa, SAU; 9 Faculty of Medicine, Tabuk University, Tabuk, SAU; 10 Radiology, Armed Forces Hospital Wadi Al Dawassir, Riyadh, SAU

**Keywords:** crohn’s disease, diagnostic accuracy, ileocolonoscopy, inflammatory bowel disease, intestinal ultrasound, meta-analysis, non-invasive monitoring, postoperative recurrence, sensitivity, specificity

## Abstract

This systematic review and meta-analysis evaluated the diagnostic performance of intestinal ultrasound (IUS) for detecting postoperative recurrence (POR) in Crohn’s disease (CD) using ileocolonoscopy as the reference standard. A comprehensive search of major databases identified 12 prospective cohort studies, including 730 patients who underwent ileocolonic or ileocecal resection. Pooled analysis demonstrated that IUS has high diagnostic accuracy, with a sensitivity of 0.87 (95% CI: 0.78-0.93), a specificity of 0.87 (95% CI: 0.77-0.93), and an overall area under the curve of 0.91, indicating strong discriminative ability. The diagnostic odds ratio was 33.91, further supporting good test performance. Sensitivity analyses confirmed the robustness of these findings, although substantial heterogeneity was observed, particularly for sensitivity. Despite variability in IUS definitions and moderate-to-serious risk of bias across studies, the results consistently showed good correlation with endoscopic findings. Potential publication bias was detected for sensitivity but not specificity. Overall, IUS appears to be a reliable, noninvasive tool for detecting POR in CD and may help reduce reliance on routine ileocolonoscopy. However, operator dependence and methodological heterogeneity should be considered when interpreting its clinical utility.

## Introduction and background

Crohn’s disease (CD) is a chronic inflammatory bowel disease (IBD) characterized by a relapsing and progressive course that can affect any part of the gastrointestinal tract [1-3]. Transmural intestinal inflammation is the primary driver of bowel damage and may lead to complications such as strictures, fistulas, and abscesses. Despite advances in medical therapy, intestinal resection remains necessary in patients with complicated or medically refractory disease, making surgery an important component of CD management [4-6].

However, surgery is not curative, and postoperative recurrence (POR) is common. Endoscopic recurrence can occur in up to 70-80% of patients within the first year after ileocolonic resection, often before clinical symptoms appear. Therefore, early postoperative monitoring is essential for guiding treatment and preventing disease progression [7,8].

Ileocolonoscopy is considered the gold standard for detecting POR. It is typically recommended 6-12 months after surgery, with recurrence graded using the Rutgeerts score to assess mucosal disease and guide treatment escalation [9]. However, repeated colonoscopy is invasive, requires bowel preparation, and may be poorly tolerated during long-term follow-up [3,5,7].

As a result, non-invasive monitoring methods have gained increasing interest. Fecal biomarkers and imaging techniques such as magnetic resonance enterography have shown potential, but their use is limited by cost, availability, and variability in accuracy [7,9].

Intestinal ultrasound (IUS) has emerged as a promising non-invasive imaging modality for IBD. It is widely available, relatively inexpensive, and well tolerated. IUS allows real-time assessment of bowel wall thickness, vascularity, and structural changes associated with inflammation. Studies have shown good correlation between IUS findings and endoscopic activity, with reported sensitivity and specificity for detecting POR reaching up to 90% [10-12].

Despite these encouraging results, evidence remains heterogeneous, and the overall diagnostic accuracy of IUS in the postoperative setting is not yet fully established. Therefore, this systematic review aims to evaluate the diagnostic performance of IUS for detecting POR in CD, using ileocolonoscopy as the reference standard, with a focus on sensitivity and specificity.

## Review

Methods

This systematic review and meta-analysis were conducted in accordance with the Preferred Reporting Items for Systematic Reviews and Meta-Analyses (PRISMA) statement [13].

Literature Search and Study Selection

A comprehensive search was performed in PubMed, Scopus, Web of Science, and Cochrane Central Register of Controlled Trials (CENTRAL) from inception to March 12, 2026. The search strategy combined terms related to CD, IUS, and POR. All retrieved records were imported into EndNote (Clarivate, London, UK) to remove duplicates (Tables 1-2).

**Table 1:** PICO framework of the systematic review This table summarizes the PICO framework used to define the eligibility criteria and research question for the systematic review. The population includes patients with CD who underwent ileocolonic or ileocecal resection and were evaluated for POR. The intervention of interest was IUS, including conventional B-mode ultrasound, Doppler ultrasound, SICUS, and CEUS. Ileocolonoscopy served as the reference standard comparator. The primary outcome was the diagnostic accuracy of IUS for detecting POR, expressed as pooled sensitivity, specificity, DOR, and AUC. PICO: population, intervention, comparator, and outcomes, IUS: intestinal ultrasound, SICUS: small intestine contrast ultrasonography, CEUS: contrast-enhanced ultrasound, DOR: diagnostic odds ratio, AUC: area under the summary receiver operating characteristic curve, CD: Crohn’s disease

PICO element	Description
Population (P)	Adult patients with CD who underwent ileocolonic or ileocecal resection and were evaluated for POR
Intervention (I)	IUS, including conventional B-mode ultrasound, Doppler ultrasound, SICUS, and CEUS
Comparator (C)	Ileocolonoscopy (reference standard for assessment of POR)
Outcome (O)	Diagnostic accuracy of IUS for detecting POR, including pooled sensitivity, specificity, DOR, and AUC

**Table 2:** Literature search strategy and study selection process This table describes the comprehensive literature search strategy and study identification process used in accordance with PRISMA guidelines. Electronic databases, including PubMed, Scopus, Web of Science, and CENTRAL, were searched from inception to March 2026. The search combined controlled vocabulary and free-text terms related to CD, IUS, and POR. No language restrictions were applied. Eligible studies included prospective and retrospective observational studies that assessed the diagnostic accuracy of IUS for POR, with ileocolonoscopy as the reference standard. Additional studies were identified through manual screening of reference lists. Study selection involved duplicate removal, title and abstract screening, and full-text review conducted independently by two reviewers using Rayyan software. PRISMA: Preferred Reporting Items for Systematic reviews and Meta-Analyses, CENTRAL: Cochrane Central Register of Controlled Trials, MeSH: Medical Subject Headings, IUS: intestinal ultrasound, SICUS: small intestine contrast ultrasonography, CEUS: contrast-enhanced ultrasound, CD: Crohn’s disease

Item	Description
Databases searched	PubMed, Scopus, Web of Science, and CENTRAL
Search period	From database inception to March 2026
Search strategy	Combination of controlled vocabulary (e.g., MeSH terms) and free-text terms related to CD, IUS, and POR
Search terms	(“Crohn disease” OR “Crohn’s disease”) AND (“intestinal ultrasound” OR “bowel ultrasound” OR “IUS” OR “small intestine contrast ultrasonography” OR “SICUS” OR “contrast-enhanced ultrasound” OR “CEUS”) AND (“postoperative recurrence” OR “post-surgical recurrence” OR “recurrence”)
Study design eligibility	Prospective cohort studies and retrospective observational studies assessing the diagnostic accuracy of IUS
Language restrictions	None applied
Additional sources	Manual screening of the reference lists of eligible articles to identify additional studies
Study selection process	Deduplication using EndNote; title/abstract screening and full-text review conducted independently by two reviewers using Rayyan software
Data extraction	Performed independently using a standardized extraction form
Disagreements resolution	Through discussion or consultation with a third reviewer

Titles and abstracts were independently screened using the Rayyan software (Rayyan Systems Inc., Cambridge, MA, USA) for systematic reviews. Full texts of potentially eligible studies were then assessed for inclusion.

Eligible studies were prospective or retrospective observational studies that evaluated the diagnostic accuracy of IUS for detecting POR in CD, with ileocolonoscopy as the reference standard. Studies were included if sufficient data were available to construct 2 × 2 contingency tables. Exclusion criteria were pediatric populations, the absence of ileocolonoscopy as the reference standard, insufficient diagnostic data, and non-original publications, such as case reports, reviews, editorials, or conference abstracts without full data.

Outcomes

The primary outcome was the diagnostic accuracy of IUS for detecting POR in CD, expressed as pooled sensitivity and specificity. Secondary outcomes included DOR and the SROC curve with AUC. Sensitivity analyses were performed using a leave-one-out approach.

POR was defined based on endoscopic findings using the Rutgeerts score, with recurrence generally defined as a Rutgeerts score ≥ i2 according to study definitions. IUS criteria for recurrence included thresholds for bowel wall thickness and additional features, such as Doppler vascularity, contrast enhancement, or lymph node enlargement, depending on the study.

Data Extraction

Data were extracted into three categories: study characteristics (author, year, country, design, and sample size), patient characteristics (age, sex, type of surgery, time since surgery, and IUS criteria), and diagnostic accuracy data (sensitivity, specificity, true positives, false positives, true negatives, and false negatives). When necessary, 2×2 contingency tables were reconstructed from available data.

Risk of Bias Assessment

Methodological quality was assessed using the Risk of Bias in Non-randomized Studies of Interventions (ROBINS-I) tool, evaluating confounding, participant selection, intervention classification, deviations from intended interventions, missing data, outcome measurement, and selective reporting [14].

Statistical Analysis

Pooled diagnostic accuracy was calculated using random-effects models. Between-study heterogeneity was assessed using the I² statistic. All analyses were performed in R (R Foundation for Statistical Computing, Vienna, Austria, https://www.R-project.org/) using the mada, meta, and metafor packages.

Results

Literature Search

**Figure 1:**
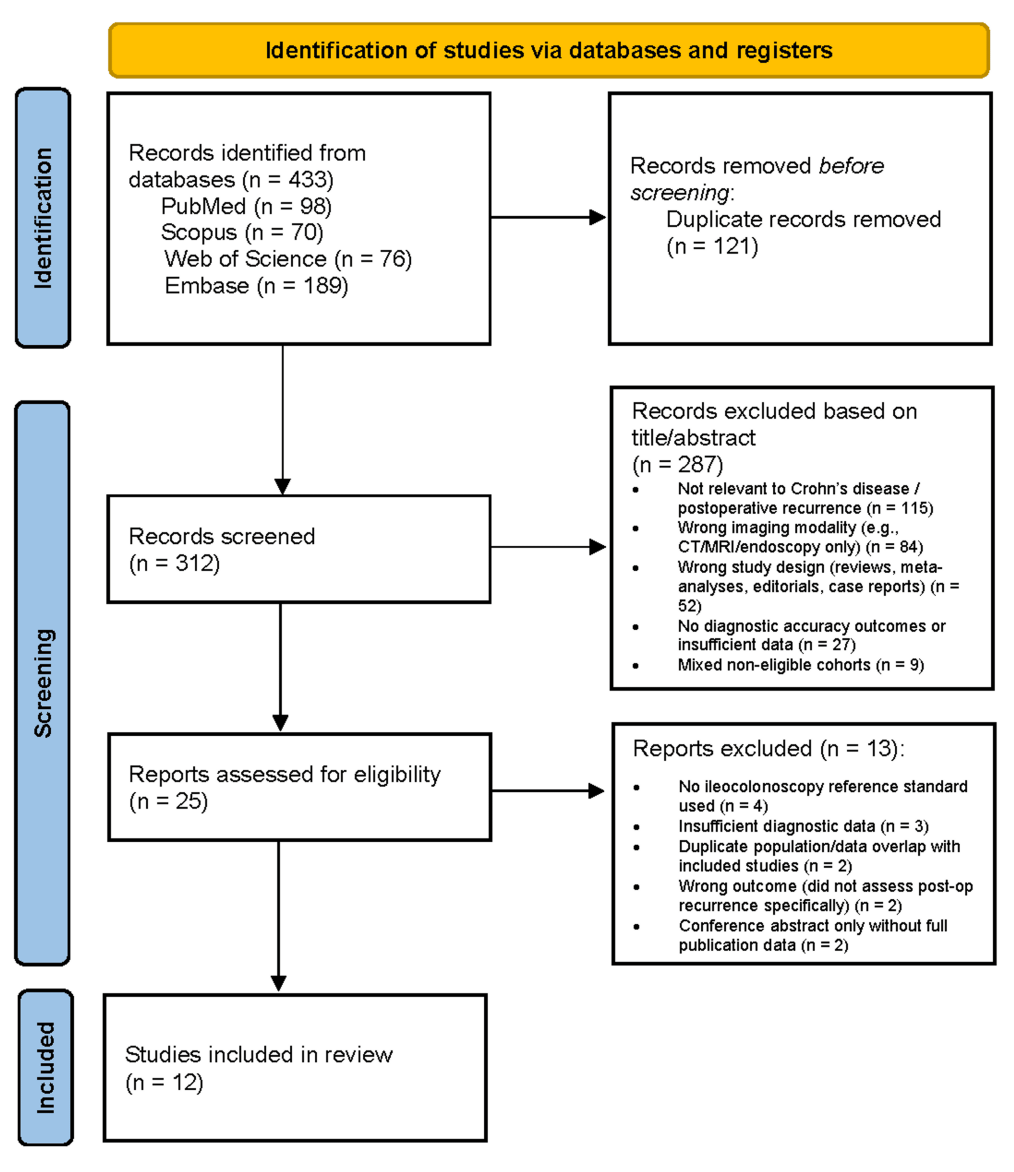
PRISMA flow diagram depicting the study selection process for the systematic review PRISMA flow diagram detailing the study selection process for this systematic review [13]. Following the identification of records through database searching and other sources, duplicates were removed, and the remaining records were screened. Full-text articles were assessed for eligibility, with exclusions documented and reasons provided. Studies meeting the inclusion criteria were included in the final synthesis. PRISMA: Preferred Reporting Items for Systematic Reviews and Meta-Analyses, CT: computed tomography, MRI: magnetic resonance imaging

The systematic search identified 433 records. After removing 121 duplicates, 312 studies were screened. Of these, 287 were excluded, and 25 full-text articles were assessed. Ultimately, 12 studies met the inclusion criteria and were included in the systematic review (Figure 1) [1-12].

Study Characteristics

Twelve prospective cohort studies published between 1986 and 2023 were included. All evaluated the diagnostic performance of IUS for detecting POR in CD. The most common surgical procedures were ileocolonic or ileocecal resection. Sample sizes ranged from 32 to 111 patients (Tables 3-4).

**Table 3:** Characteristics and diagnostic findings of studies evaluating IUS for POR in CD This table summarizes the characteristics, index test protocols, reference standards, key findings, and conclusions of the included prospective cohort studies assessing the diagnostic performance of IUS for detecting POR in CD. All studies used ileocolonoscopy as the reference standard, with recurrence generally defined according to endoscopic findings. Index test protocols varied across studies and included conventional ultrasound, SICUS, Doppler ultrasound, and CEUS, with differing thresholds for BWT and additional parameters such as vascularity and lymph node assessment. Sample size marked with an asterisk (*) indicates values as reported in the original study. IUS: intestinal ultrasound, POR: postoperative recurrence, BWT: bowel wall thickness, SICUS: small intestine contrast ultrasonography, CEUS: contrast-enhanced ultrasound, BS: bowel sonography, OCBS: oral contrast bowel sonography, CD: Crohn’s disease

Study ID	Country	Design	Sample size	Index test protocol	Reference standard	Key findings	Conclusion
Furfaro et al. [1]	Italy	Prospective cohort	91	Bowel ultrasound within 1 year; assessment included mesenteric lymph nodes as a diagnostic parameter	Ileocolonoscopy	Presence of lymph nodes and bowel wall changes correlated with endoscopic recurrence.	IUS can effectively detect POR and inflammatory changes.
Andreoli et al. [2]	Italy	Prospective cohort	41	Conventional abdominal ultrasound measuring bowel wall thickness (>5 mm)	Ileocolonoscopy	Ultrasound detected bowel wall thickening associated with POR, with high diagnostic agreement with endoscopy.	Ultrasound may represent a useful noninvasive tool for detecting postoperative Crohn’s recurrence.
Calabrese et al. [3]	Italy	Prospective cohort	72	SICUS; recurrence defined by BWT >3 mm or dilation/stricture	Ileocolonoscopy	SICUS demonstrated high diagnostic accuracy in identifying POR and assessing disease severity.	SICUS is an effective technique for detecting and grading POR in CD.
Castiglione et al. [4]	Italy	Prospective cohort	40	Standard BS and OCBS; study-specific cutoff values applied	Ileocolonoscopy	Increased bowel wall thickness correlated with endoscopic recurrence severity; ultrasound showed good diagnostic performance.	Ultrasound can reliably identify POR and may help stratify disease severity.
DiCandio et al. [5]	Italy	Prospective cohort	32	Sonography for POR	Ileocolonoscopy	Contrast-enhanced ultrasound improved visualization of intestinal vascularity and inflammatory activity in recurrent CD.	Contrast-enhanced ultrasound may enhance diagnostic accuracy in POR.
Macedo et al. [6]	Portugal	Prospective cohort	39	IUS (Eco-PD); abnormal if BWT >3 mm and/or Limberg score >1	Ileocolonoscopy	Ultrasound parameters showed moderate sensitivity and good specificity for detecting POR.	IUS is a useful noninvasive monitoring tool after Crohn’s surgery.
Martínez et al. [7]	Spain	Prospective cohort	108	B-mode ultrasound, color Doppler, and CEUS; recurrence defined as wall thickness ≥3 mm	Ileocolonoscopy	Ultrasound findings correlated well with the Rutgeerts endoscopic score.	IUS is reliable for postoperative monitoring and detection of recurrence.
Onali et al. [8]	Italy	Prospective cohort	58	SICUS	Ileocolonoscopy	SICUS detected POR with high sensitivity and improved visualization of small bowel lesions.	SICUS represents an accurate imaging technique for detecting recurrence after surgery.
Pallotta et al. [9]	Italy	Prospective cohort	111	SICUS; main cutoff: ileocolic anastomosis wall thickness >3.5 mm (score 0 vs 1–4)	Ileocolonoscopy	Bowel wall thickness measured by ultrasound strongly correlated with endoscopic recurrence.	Ultrasound bowel wall measurement is a useful indicator of postoperative Crohn’s recurrence.
Paredes et al. [10]	Spain	Prospective cohort	33	Ultrasound combined with Doppler	Ileocolonoscopy	Doppler vascularity and bowel wall thickening improved the identification of inflammatory recurrence.	Doppler ultrasound may enhance the detection of active POR.
Paredes et al. [11]	Spain	Prospective cohort	60	Ultrasound combined with CEUS	Ileocolonoscopy	CEUS improved sensitivity and overall diagnostic accuracy compared with conventional ultrasound.	Contrast-enhanced ultrasound significantly improves diagnostic performance for POR.
Rispo et al. [12]	Italy	Prospective cohort	45	Bowel sonography	Ileocolonoscopy	Bowel sonography demonstrated 79% sensitivity and 95% specificity for detecting postsurgical recurrence.	Bowel sonography is an accurate and noninvasive alternative for detecting POR.

**Table 4:** Patient characteristics and ultrasound criteria of included studies This table summarizes patient demographics, surgical characteristics, time since surgery, and ultrasound diagnostic criteria used in the included prospective cohort studies evaluating IUS for detecting POR in CD. Reported variables include mean age with SD, sex distribution, type of surgical procedure, and timing of ultrasound assessment following surgery. Ultrasound cut-off definitions varied across studies and were primarily based on BWT, with additional parameters including luminal dilation, strictures, Doppler vascularity (Limberg score), CEUS enhancement, and the presence of mesenteric lymph nodes. Time since surgery is reported as mean, median, range, or IQR as provided in the original studies. SD: standard deviation, IQR: interquartile range, BWT: bowel wall thickness, CEUS: contrast-enhanced ultrasound, IUS: intestinal ultrasound, POR: postoperative recurrence, CD: Crohn’s disease

Study ID	Age (mean ± SD)	Male, n (%)	Type of surgery	Time since surgery	Ultrasound cut-off
Furfaro et al. [1]	39.3 ± 16.3	55 (60%)	Ileocolonic resection with ileocolonic anastomosis	Median 6.5 months	Presence of mesenteric lymph nodes
Andreoli et al. [2]	42.4 ± 16.5	26 (63.4%)	Ileocolonic resection with anastomosis	Mean 35.4 months (range 3–105)	BWT >5 mm
Calabrese et al. [3]	44.3 ± 14.3	34 (47.3%)	Ileocolonic resection	Median 18 months (range 3–396)	BWT >3 mm ± dilation >25 mm or stricture <10 mm
Castiglione et al. [4]	43.8 ± 14.8	24 (55.8%)	Ileocolonic resection	12 months	Standard bowel sonography
DiCandio et al. [5]	41.5 ± 12.0	24 (75%)	Ileocolonic resection (28), ileorectal anastomosis (4)	Mean 4.5 years (range 1–12)	BWT >5 mm
Macedo et al. [6]	43.5 ± 15.3	14 (35.9%)	Ileocecal resection	Median 9 years (IQR 3–12)	BWT >3 mm and/or Limberg Doppler score >1
Martínez et al. [7]	43.7 ± 14.0	55 (50.9%)	Ileocaecal resection (67.6%), ileocolonic resection (32.4%)	Mean 6 years (range 3 months–30 years)	Recurrence defined as BWT ≥3 mm
Onali et al. [8]	38.8 ± 13.3	12 (48%)	Ileocolonic resection	12 months	BWT >3 mm
Pallotta et al. [9]	46.2 ± 14.0	37 (63.7%)	Ileal resection with ileocolonic anastomosis	6–24 months follow-up	Ileocolic anastomosis wall thickness >3.5 mm
Paredes et al. [10]	41.2 ± 11.3	22 (57.9%)	Ileocolic resection with anastomosis	Median 87.7 ± 75.4 months	BWT >3 mm and/or positive Doppler flow
Paredes et al. [11]	39.0 ± 11.3	32 (53.3%)	Ileocolic resection with ileocolonic anastomosis	Median 60 months	BWT >3 mm plus CEUS enhancement ≥34.5%
Rispo et al. [12]	33.5 ± 9.5	25 (55.5%)	Ileocolonic anastomosis (80%), ileotransverse anastomosis (20%); stricturoplasty 11%	12 months	BWT ≤3 mm considered normal

Ileocolonoscopy was used as the reference standard in all studies. POR was primarily defined using the Rutgeerts score and assessed during follow-up periods ranging from six months to several years.

Risk of Bias Assessment

Most studies showed a moderate-to-serious risk of bias, mainly due to confounding and participant selection. Index test classification and outcome measurement domains generally showed low risk of bias (Table 5).

**Table 5:** Risk of bias assessment of included studies using the ROBINS-I tool This table presents the methodological quality assessment of the included studies using the ROBINS-I tool [14]. Each study was evaluated across seven bias domains: D1 (bias due to confounding), D2 (bias in selection of participants), D3 (bias in classification of interventions), D4 (bias due to deviations from intended interventions), D5 (bias due to missing data), D6 (bias in measurement of outcomes), and D7 (bias in selection of the reported result). The overall risk-of-bias judgment for each study reflects the highest level of bias identified across all domains, categorized as low, moderate, or serious. ROBINS-I: Risk of Bias in Non-randomized Studies of Interventions

Study ID	D1	D2	D3	D4	D5	D6	D7	Overall
Furfaro et al. [1]	Moderate	Moderate	Low	Low	Moderate	Low	Moderate	Moderate
Andreoli et al. [2]	Moderate	Moderate	Low	Low	Moderate	Low	Moderate	Moderate
Calabrese et al. [3]	Serious	Moderate	Low	Moderate	Low	Moderate	Moderate	Serious
Castiglione et al. [4]	Moderate	Moderate	Low	Low	Moderate	Low	Moderate	Moderate
DiCandio et al. [5]	Moderate	Moderate	Low	Low	Low	Moderate	Moderate	Moderate
Macedo et al. [6]	Moderate	Moderate	Low	Low	Low	Low	Moderate	Moderate
Martínez et al. [7]	Serious	Moderate	Low	Moderate	Moderate	Low	Moderate	Serious
Onali et al. [8]	Moderate	Moderate	Low	Moderate	Serious	Low	Moderate	Serious
Pallotta et al. [9]	Serious	Moderate	Low	Low	Moderate	Low	Moderate	Serious
Paredes et al. [10]	Moderate	Serious	Low	Low	Serious	Low	Moderate	Serious
Paredes et al. [11]	Moderate	Moderate	Low	Low	Low	Low	Moderate	Moderate
Rispo et al. [12]	Moderate	Moderate	Low	Low	Moderate	Low	Moderate	Moderate

Pooled Diagnostic Accuracy

**Figure 2:**
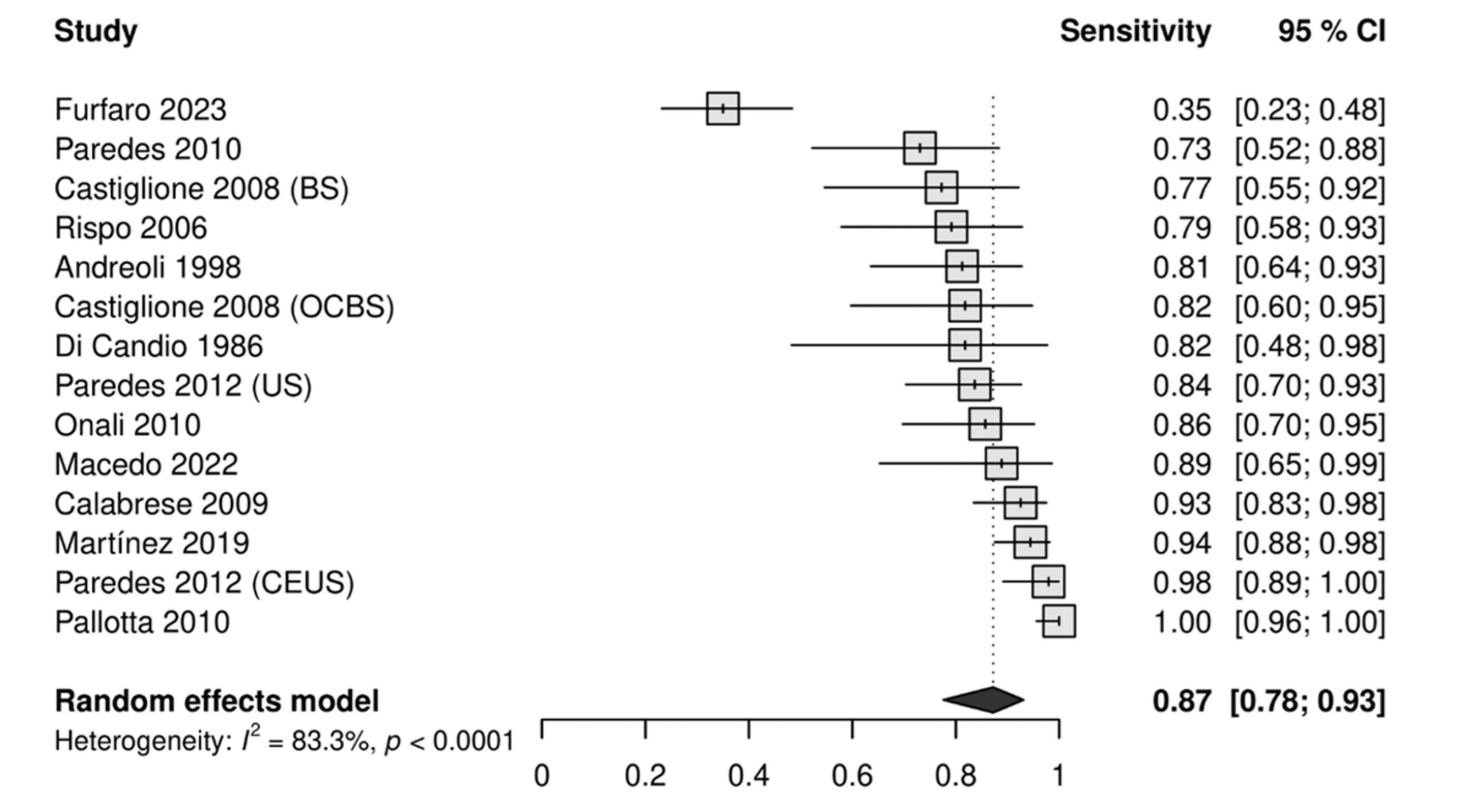
Forest plot of pooled sensitivity of IUS for detecting POR in CD Forest plot displaying sensitivity estimates of IUS for detecting POR in CD across included studies (Furfaro et al., 2023 [1]; Andreoli et al., 1998 [2]; Calabrese et al., 2009 [3]; Castiglione et al., 2008 [4]; DiCandio et al., 1986 [5]; Macedo et al., 2022 [6]; Martínez et al., 2019 [7]; Onali et al., 2010 [8]; Pallotta et al., 2010 [9]; Paredes et al., 2010 [10]; Paredes et al., 2013 [11]; Rispo et al., 2006 [12]). Each square represents an individual study estimate, with horizontal lines indicating 95% CI and square size reflecting study weight. The pooled sensitivity is presented as a diamond based on a random-effects model. Heterogeneity among studies is quantified using the I² statistic. IUS: intestinal ultrasound, POR: postoperative recurrence, CD: Crohn’s disease, CI: confidence interval

**Figure 3:**
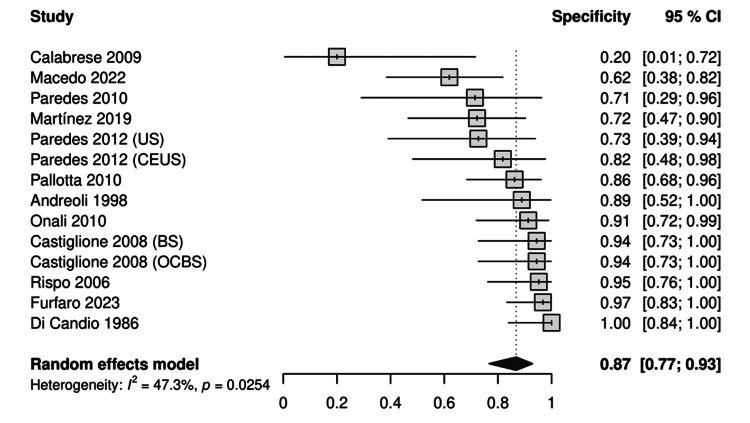
Forest plot of pooled specificity of IUS for detecting POR in CD Forest plot displaying specificity estimates of IUS for detecting POR in CD across included studies (Furfaro et al., 2023 [1]; Andreoli et al., 1998 [2]; Calabrese et al., 2009 [3]; Castiglione et al., 2008 [4]; DiCandio et al., 1986 [5]; Macedo et al., 2022 [6]; Martínez et al., 2019 [7]; Onali et al., 2010 [8]; Pallotta et al., 2010 [9]; Paredes et al., 2010 [10]; Paredes et al., 2013 [11]; Rispo et al., 2006 [12]). Each square represents an individual study estimate, with horizontal lines indicating 95% CI and square size reflecting study weight. The pooled specificity is presented as a diamond based on a random-effects model. Heterogeneity among studies is quantified using the I² statistic. IUS: intestinal ultrasound, US: ultrasound, CEUS: contrast-enhanced ultrasound, BS: bowel sonography, OCBS: oral contrast bowel sonography, POR: postoperative recurrence, CD: Crohn’s disease, CI: confidence interval

**Figure 4:**
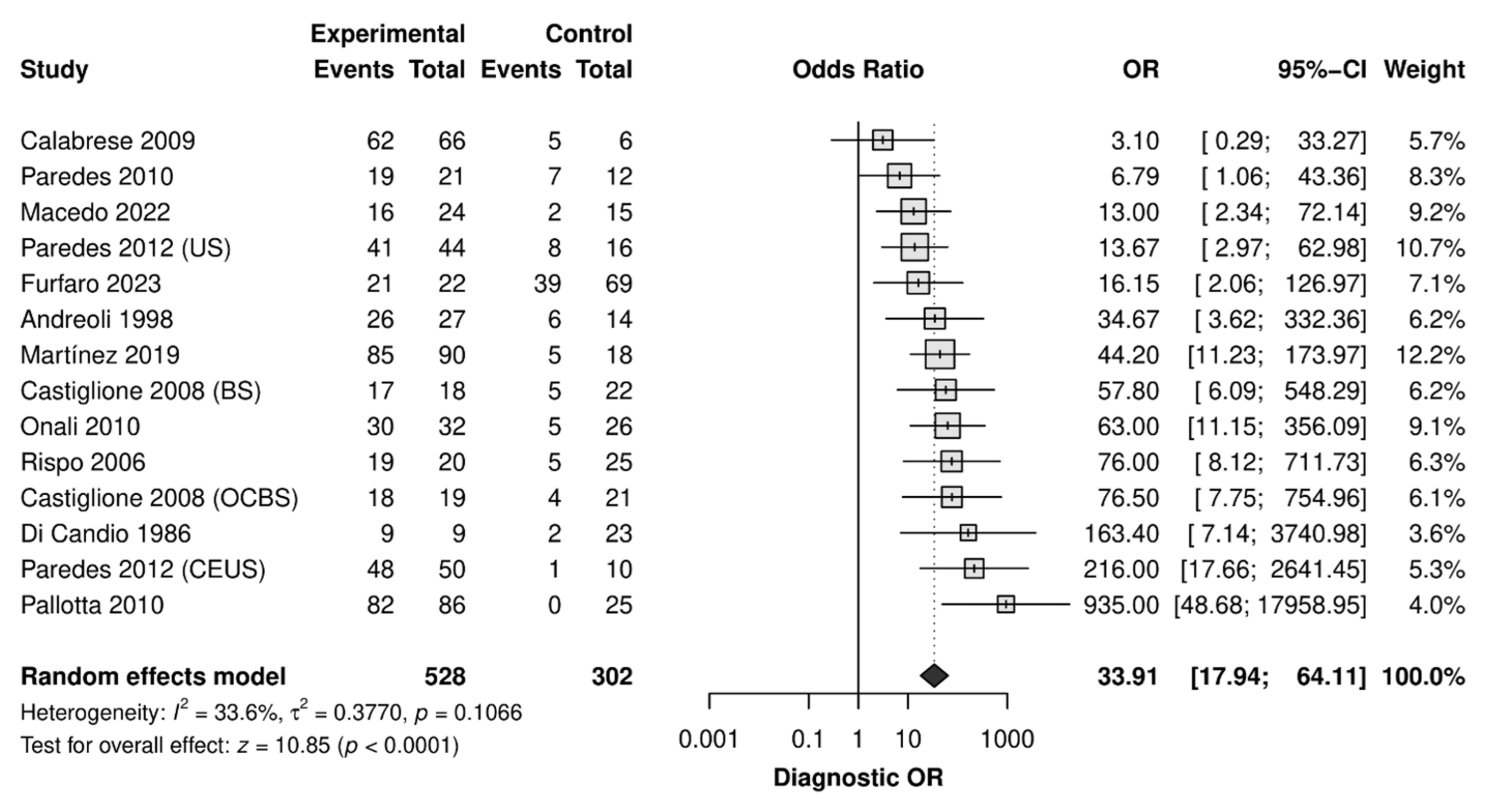
Forest plot of DORs ratio of IUS for detecting POR in CD Forest plot displaying the DORs for IUS in detecting POR in CD across included studies (Furfaro et al., 2023 [1]; Andreoli et al., 1998 [2]; Calabrese et al., 2009 [3]; Castiglione et al., 2008 [4]; DiCandio et al., 1986 [5]; Macedo et al., 2022 [6]; Martínez et al., 2019 [7]; Onali et al., 2010 [8]; Pallotta et al., 2010 [9]; Paredes et al., 2010 [10]; Paredes et al., 2013 [11]; Rispo et al., 2006 [12]). Each square represents an individual study’s DOR, with horizontal lines indicating 95% CI and square size reflecting study weight. The pooled DOR is presented as a diamond based on a random-effects model. A logarithmic scale is typically used for DOR, and heterogeneity among studies is quantified using the I² statistic. DORs: diagnostic odds ratios, IUS: intestinal ultrasound, US: ultrasound, BS: bowel sonography, OCBS: oral contrast bowel sonography, CEUS: contrast-enhanced ultrasound, POR: postoperative recurrence, CD: Crohn’s disease, CI: confidence interval, OR: odds ratio

A total of 12 studies, including 730 patients, were analyzed. The pooled sensitivity of IUS for detecting POR was 0.87 (95% CI: 0.78-0.93), with substantial heterogeneity (I² = 83.3%, p < 0.0001). The pooled specificity was 0.87 (95% CI: 0.77-0.93), with moderate heterogeneity (I² = 47.3%, p = 0.025). Overall diagnostic performance was high, with an AUC of 0.91. The DOR was 33.91 (95% CI: 17.94-64.11; p < 0.0001), indicating good discriminatory ability of IUS. Moderate heterogeneity was observed (I² = 33.6%, τ² = 0.377, p = 0.106) (Figures 2-4).

Sensitivity Analysis

Leave-one-out analyses showed stable results. Sensitivity ranged from 0.84 to 0.89, and specificity ranged from 0.85 to 0.88. Exclusion of a single influential study reduced heterogeneity in sensitivity from 83.8% to 34.4%, while specificity remained stable.

Publication Bias

**Figure 5:**
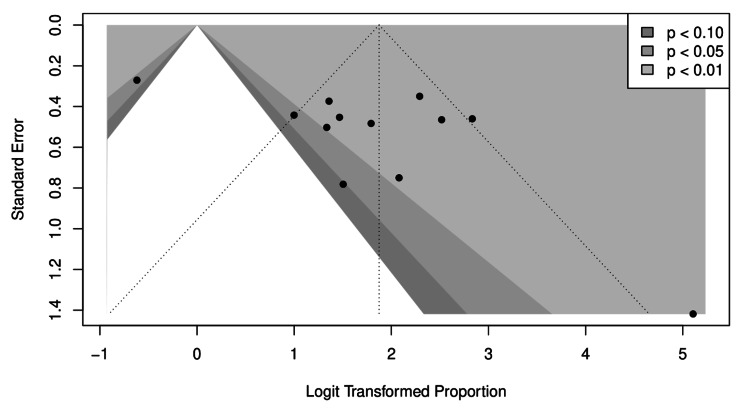
Funnel plot assessing publication bias for sensitivity of IUS in detecting POR in CD Funnel plot evaluating potential publication bias for sensitivity estimates of IUS in detecting POR in CD across included studies (Furfaro et al., 2023 [1]; Andreoli et al., 1998 [2]; Calabrese et al., 2009 [3]; Castiglione et al., 2008 [4]; DiCandio et al., 1986 [5]; Macedo et al., 2022 [6]; Martínez et al., 2019 [7]; Onali et al., 2010 [8]; Pallotta et al., 2010 [9]; Paredes et al., 2010 [10]; Paredes et al., 2013 [11]; Rispo et al., 2006 [12]). Each point represents an individual study plotted by its sensitivity estimate against the corresponding standard error. Visual inspection suggests asymmetry, indicating possible publication bias or small-study effects, which is supported by Egger’s test. IUS: intestinal ultrasound, POR: postoperative recurrence, CD: Crohn’s disease

**Figure 6:**
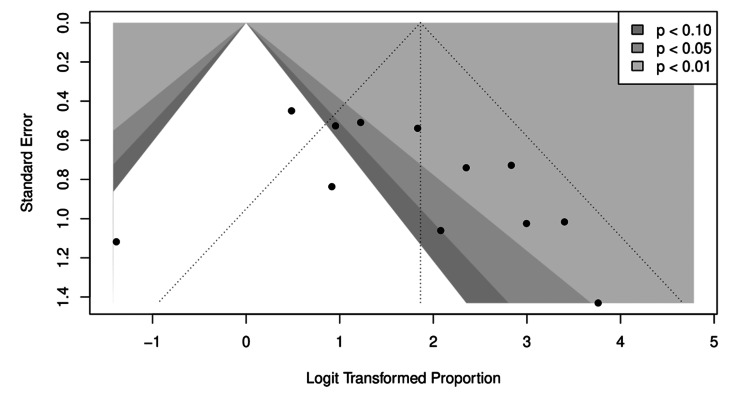
Funnel plot assessing publication bias for specificity of IUS in detecting POR in CD Funnel plot evaluating potential publication bias for specificity estimates of IUS in detecting POR in CD across included studies (Furfaro et al., 2023 [1]; Andreoli et al., 1998 [2]; Calabrese et al., 2009 [3]; Castiglione et al., 2008 [4]; DiCandio et al., 1986 [5]; Macedo et al., 2022 [6]; Martínez et al., 2019 [7]; Onali et al., 2010 [8]; Pallotta et al., 2010 [9]; Paredes et al., 2010 [10]; Paredes et al., 2013 [11]; Rispo et al., 2006 [12]). Each point represents an individual study plotted by its specificity estimate against the corresponding standard error. The distribution appears relatively symmetrical, suggesting no significant publication bias, consistent with Egger’s test findings. IUS: intestinal ultrasound, POR: postoperative recurrence, CD: Crohn’s disease

Funnel plot asymmetry suggested possible publication bias for sensitivity, supported by Egger’s test (p = 0.0377). No significant publication bias was observed for specificity (Egger’s test p = 0.1505) (Figures 5-6).

Discussion

Comparative Performance Across Studies

Across the 12 included studies, IUS demonstrated consistently good diagnostic performance for detecting POR in CD, although variability between studies was observed. Pooled estimates showed high sensitivity and specificity, but individual studies reported a range of values reflecting differences in ultrasound technique, operator experience, and definitions of recurrence [1-12].

Studies such as Pallotta et al. and Rispo et al. reported particularly high specificity, suggesting that IUS is highly reliable for ruling out recurrence when normal findings are present [9,12]. In contrast, sensitivity varied more widely, which is consistent with earlier prospective studies showing operator dependence and methodological variability in ultrasonographic assessment of postoperative CD [2,5,8].

The substantial heterogeneity observed in this meta-analysis, particularly in sensitivity, is also consistent with findings from comparative longitudinal studies, in which ultrasound performance varied with timing after surgery and imaging protocol [8,10]. Differences in BWT thresholds, use of Doppler signals, and contrast-enhanced techniques further contributed to variability between studies [3,7,11].

Pathophysiological Basis of IUS Findings

The diagnostic accuracy of IUS is supported by the transmural nature of CD, which leads to structural and vascular changes detectable on ultrasound [2,9]. Bowel wall thickening reflects inflammatory infiltration and edema, whereas Doppler vascularity and contrast enhancement reflect active inflammation [7,11].

Several studies demonstrated a strong correlation between sonographic findings and endoscopic recurrence, as graded by the Rutgeerts score, reinforcing the biological plausibility of IUS as a surrogate marker of mucosal disease activity [8,10]. In particular, Calabrese et al. demonstrated a clear correlation between ultrasound findings and severity of POR, highlighting its role in disease stratification [3].

POR typically begins at or near the ileocolonic anastomosis, a region easily accessible to ultrasound, which likely contributes to the high diagnostic yield observed across studies [9,12].

Comparison With Included Study Evidence

Early foundational work by DiCandio et al. first demonstrated the feasibility of ultrasound in detecting POR in CD, establishing bowel wall thickening as a key marker of recurrence [5]. Subsequent studies expanded on this by incorporating contrast-enhanced and Doppler techniques, improving diagnostic performance [4,7,11].

Castiglione et al. showed that oral contrast-enhanced ultrasound improves POR grading compared with standard sonography, suggesting that contrast-enhanced ultrasound enhances lesion detection and characterization [4]. Similarly, Martínez et al. and Paredes et al. demonstrated that contrast-enhanced ultrasound provides improved sensitivity and correlation with endoscopic activity [7,11].

More recent prospective studies, including Furfaro et al. and Macedo et al., confirmed that modern IUS techniques maintain high diagnostic accuracy and strong correlation with endoscopic recurrence in real-world postoperative surveillance settings [1,6].

Comparison With Conventional Ultrasound Approaches

Conventional bowel sonography has been shown to detect POR with reasonable accuracy, particularly when bowel wall thickness is used as the primary diagnostic criterion [2,9]. However, several studies indicate that adding Doppler flow assessment or contrast enhancement improves sensitivity and overall diagnostic performance [7,11].

Rispo et al. demonstrated that bowel sonography can achieve high specificity for detecting recurrence, supporting its role as a noninvasive surveillance tool [12]. Similarly, Pallotta et al. showed that bowel wall measurement alone can reliably predict recurrence, although accuracy improves when combined with additional imaging features [9].

Clinical Implications

The findings of this review support the integration of IUS into postoperative surveillance strategies for CD. Given its noninvasive nature and strong correlation with endoscopic recurrence, IUS may serve as a first-line screening tool after ileocolonic resection [1,6,8].

IUS could potentially reduce the need for routine ileocolonoscopy by identifying patients who require further endoscopic evaluation based on abnormal findings. This approach is particularly relevant given the invasive nature and cost of repeated endoscopy in long-term follow-up [9,10].

Furthermore, IUS may be useful in guiding early therapeutic escalation in patients with subclinical recurrence, as imaging-detected recurrence often precedes clinical symptoms [8,10].

Limitations

Despite promising results, several limitations must be considered. Most included studies were observational and were subject to moderate to serious risk of bias, particularly in the confounding and participant selection domains, as assessed by ROBINS-I [14].

Operator dependence remains a major limitation of IUS, contributing to variability in diagnostic performance across studies [2,6]. Additionally, heterogeneity in ultrasound protocols, including differences in BWT cut-offs, Doppler usage, and contrast enhancement techniques, further limited comparability between studies [3,7,11].

Definitions of POR were also not fully standardized, although most studies used ileocolonoscopy as the reference standard [8,10]. Finally, many studies were conducted in specialized centers, which may limit generalizability to routine clinical practice [1,6].

## Conclusions

IUS demonstrates good diagnostic performance for detecting POR in CD, with balanced sensitivity and specificity and strong overall discriminative ability. It may serve as a useful non-invasive tool for postoperative monitoring and potentially reduce the need for routine ileocolonoscopy, improving patient experience. However, variability across studies and operator dependence should be considered when interpreting results.
